# Broadband Flexible Metasurface for SAR Imaging Cloaking

**DOI:** 10.3390/ma18173969

**Published:** 2025-08-25

**Authors:** Bo Yang, Hui Jin, Chaobiao Chen, Peixuan Zhu, Siqi Zhang, Rongrong Zhu, Bin Zheng, Huan Lu

**Affiliations:** 1 State Key Laboratory of Extreme Photonics and Instrumentation, ZJU-Hangzhou Global Scientific and Technological Innovation Center, Zhejiang University, Hangzhou 310027, China; 18867645958@163.com (B.Y.); 22231011@zju.edu.cn (C.C.); peixuanzhu@zju.edu.cn (P.Z.); sjgqzhang@outlook.com (S.Z.); luhuan123@zju.edu.cn (H.L.); 2 International Joint Innovation Center, The Electromagnetics Academy at Zhejiang University, Zhejiang University, Haining 314400, China; 3 Zhejiang Key Laboratory of Intelligent Electromagnetic Control and Advanced Electronic Integration, Jinhua Institute of Zhejiang University, Zhejiang University, Jinhua 321099, China; 4 Sussex Artificial Intelligence Institute, Zhejiang Gongshang University, Hangzhou 310018, China; 5 School of Information and Electrical Engineering, Hangzhou City University, Hangzhou 310015, China; rorozhu@zju.edu.cn

**Keywords:** metasurface, flexible materials, SAR imaging cloak

## Abstract

Most electromagnetic invisibility devices are designed while relying on rigid structures, which have limitations in adapting to complex curved surfaces and dynamic deployment. In contrast, flexible invisibility structures have great application value due to their bendable and easy-to-fit characteristics. In this paper, we propose a flexible metasurface suitable for broadband SAR (Synthetic Aperture Radar) imaging invisibility, which realizes multi-domain joint regulation of electromagnetic waves by designing two subwavelength unit structures with differentiated reflection characteristics and combining array inverse optimization methods. The metasurface employs a sponge-like dielectric substrate and integrates resistive ink to construct a resonant structure, which can suppress electromagnetic scattering through joint phase and amplitude modulation, achieving low detectability of targets in UAV (Unmanned Aerial Vehicle) detection scenarios. Indoor microwave anechoic chamber tests and outdoor UAV-borne SAR experiments verify its stable invisibility performance in a wide frequency band, providing theoretical and experimental support for the application of flexible metasurfaces in dynamic electromagnetic detection countermeasures.

## 1. Introduction

As an artificially designed subwavelength electromagnetic (EM) structure, metasurfaces have attracted extensive attention due to their excellent flexibility and controllability in EM wave manipulation [1,2,3,4,5]. Their unique subwavelength characteristics enable precise regulation of EM parameters such as amplitude, phase, and polarization, providing a foundation for diverse functional applications [6,7,8,9]. To date, metasurfaces have been widely explored in fields including beam focusing and control [10,11,12,13], polarization conversion [14,15], and EM cloaking [16,17,18,19]. Among these, EM cloaking stands out as one of the most representative applications, aiming to reduce target detectability by modulating EM wave scattering properties [20,21,22]. This can be achieved through multiple mechanisms: scattering cancelation, which offsets the original scattering field via induced secondary radiation [21,22]; carpet cloaking, which bends EM waves around the target to “hide” its presence^16^; and absorbing cloaking, which dissipates incident EM energy to suppress reflections [23,24,25]. Among these, absorbing cloaking has become a mainstream technical approach due to its straightforward principle, low engineering implementation difficulty, and effective reduction of radar cross section (RCS), especially in scenarios requiring broadband and low reflectivity.

However, existing research on absorbing cloaking mostly focuses on single-amplitude modulation [23,24,25]. By designing materials or structures to absorb EM wave energy, these studies achieve cloaking effects but are limited by the single modulation dimension, making it difficult to maintain efficiency and stability across a wide frequency band. In contrast, multi-domain modulation (combining amplitude and phase) could break through this limitation, but it involves complex coupling of EM parameters, leading to high design difficulty and relatively few related studies. Additionally, traditional cloaking devices are mostly fabricated on rigid substrates [26,27,28], resulting in poor conformal ability. This restricts their application in scenarios requiring adaptation to curved targets or flexible carriers (e.g., field temporary facilities and wearable devices), highlighting the need for flexible metasurface-based cloaking solutions. Therefore, determining how to design an invisible structure based on a flexible substrate and simultaneously achieve effective regulation of amplitude and phase by combining intelligent strategies [29,30,31,32,33] is of great significance in practical applications.

To address the above issues, this paper presents a metasurface cloaking device based on flexible substrates with joint amplitude-phase regulation. The metasurface consists of two resonant structures with differentiated reflection characteristics and realizes multi-domain coordinated regulation of EM waves through inverse design methods to optimize array arrangement. Its substrate uses flexible sponge material, integrating resistive ink and metal layers to construct resonant structures. The fabricated metasurface has both ultra-flexible characteristics and broadband absorption capacity, showing excellent EM wave absorption performance in microwave anechoic chamber tests. Meanwhile, outdoor UAV-borne SAR tests verify its effective cloaking effect in the X-band. This work provides a new design idea for broadband EM cloaking in flexible scenarios and is of great significance for promoting the engineering application of metasurfaces in dynamic detection countermeasures.

## 2. Methods

**Figure 1:**
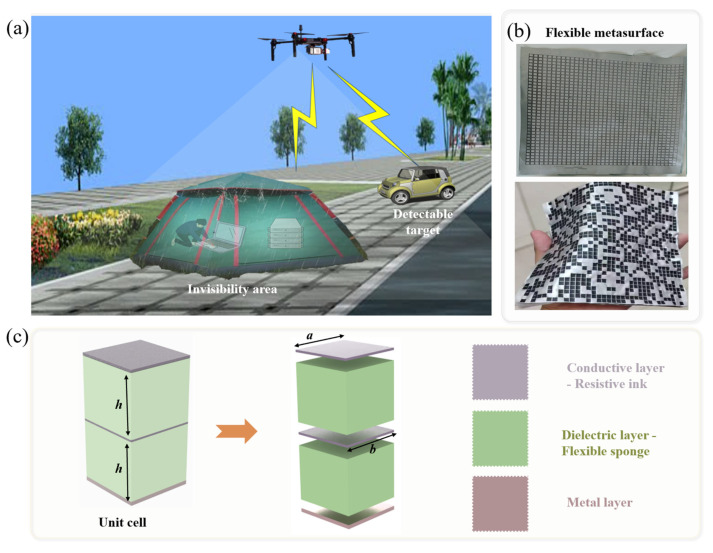
Application scenarios and basic composition of the flexible SAR imaging cloaking metasurface. (**a**) Potential application scenario. (**b**) Physical image of the flexible metasurface. (**c**) Basic unit cell.

Figure 1 illustrates the potential application scenarios and structural composition of the flexible metasurface. Figure 1a presents a UAV EM detection countermeasure scenario based on the flexible metasurface: the UAV is equipped with EM sensing loads and emits EM waves to the ground to perform reconnaissance tasks; the vehicle on the right is a conventional detectable target without EM cloaking measures, whose EM scattering characteristics are not regulated, making its echo signals easy to be captured and identified by the UAV; the “Invisibility area” (tent structure) on the left, constructed by the flexible metasurface, can achieve precise regulation of the phase, amplitude and polarization state of the incident EM waves by virtue of subwavelength microstructures. On one hand, it can guide EM waves to diffract around the tent as well as the personnel and equipment inside to suppress scattered echoes; on the other hand, it can simulate the EM response of background environments such as the ground and vegetation, reducing the difference in EM characteristics between the tent and the surrounding environment, thus making it difficult for the UAV to distinguish the target from the background from the echoes. Figure 1b shows the sample of the flexible metamaterial, which uses a two-layer structure design with a flexible foam medium supporting between the layers. Figure 1c shows the composition of the basic unit of the metasurface, which consists of two dielectric layers, two flexible ink layers and one total reflection metal layer.

The scenario in Figure 1 intuitively demonstrates the application logic of the flexible metasurface in anti-UAV detection: by utilizing its bendable property and easy adaptability to complex shapes, it breaks through the deployment limitations of traditional rigid metasurfaces, constructs a low-detectability protective layer for ground temporary facilities such as field operation tents, and meets the requirements of flexible and lightweight EM cloaking. Additionally, it provides a visual reference for the research on the EM wave regulation mechanism and engineering application of the flexible metasurface.

When a reflective metasurface contains two types of unit cell structures (unit cell 1 and unit cell 2), the reduction value $\sigma_{r}$ of the radar cross section in the vertical exit direction when EM waves are incident vertically is determined by Equation (1) [34]

(1) $$\sigma_{r}=20\log\left[\left(\frac{n_{1}}{n_{1}+n_{2}}A_{1}e^{-i\varphi_{1}}\right)+\left(\frac{n_{2}}{n_{1}+n_{2}}A_{2}e^{-i\varphi_{2}}\right)\right]$$

Among them, $n_{1}$ and $n_{2}$ are the quantities of unit cell 1 and unit cell 2 in the metasurface, respectively; $A_{1}$ and $A_{2}$ are the reflectivity amplitudes of unit cell 1 and unit cell 2, respectively; $\varphi_{1}$ and $\varphi_{2}$ are the reflectivity phases of unit cell 1 and unit cell 2, respectively. Let the quantity proportion of unit cell 1 be ξ and the phase difference between the two unit structures be $\varphi_{dif}$, then ξ and $\varphi_{dif}$ can be expressed as [32]:

(2) $$\xi =\frac{n_{1}}{n_{1}+n_{2}}$$

(3) $$\varphi_{dif}=\varphi_{1}-\varphi_{2}$$

If the two types of unit cells have no wave absorption effect, i.e., $A_{1}$ = $A_{2}$ = 1, the RCS reduction value $\sigma_{r}$ is only determined by the quantity proportion ξ of unit cell 1 and the phase difference $\varphi_{dif}$ between the two structures. When the phase difference is close to 180°, even if the unit cells themselves have no wave absorption effect, the metasurface after array arrangement can still achieve an RCS reduction of more than 10 dB; however, to achieve broadband amplitude regulation, the amplitude difference between unit cells needs to be considered.

**Figure 2:**
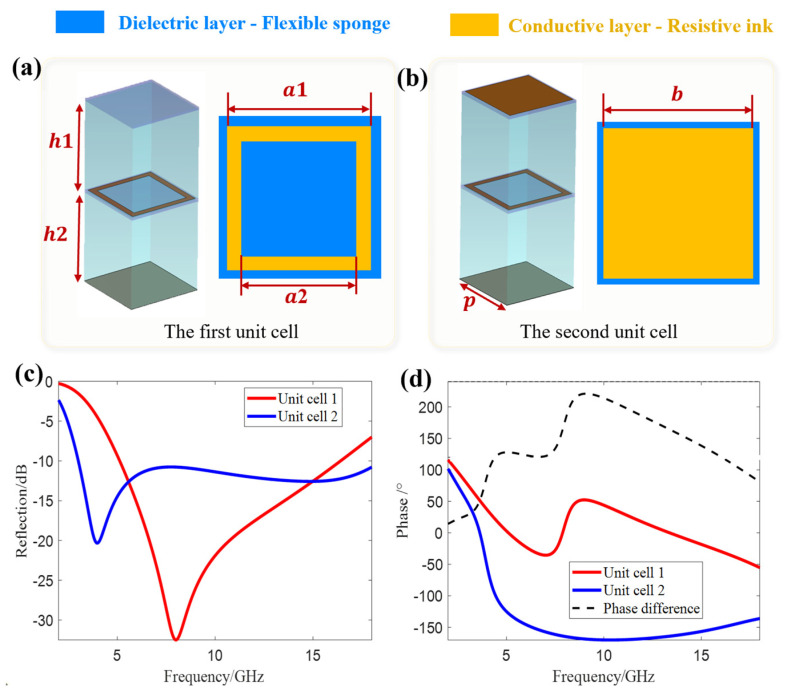
Two types of basic unit cells and their reflection characteristics. (**a**) The first unit cell. (**b**) The second unit cell. (**c**) Amplitude responses of the two unit cells. (**d**) Phase responses and phase difference of the two unit cells.

To this end, two types of unit cells with different reflectivity amplitudes and phase characteristics are designed (Figure 2). Figure 2a,b show the structures of unit cell 1 and unit cell 2, respectively. Both unit cells have a side length p = 5 mm and a dielectric thickness h1 = h1 = 6 mm. Figure 2c,d present their amplitude responses, phase responses, and phase difference.

Both unit cells employ a stacked structure of two sub-metasurfaces, where each sub-metasurface is composed of cloth containing resistive ink and dielectric material (note: the upper sub-metasurface of unit cell 1 is pure flexible cloth without resistive ink). To achieve reflectivity difference, the resistive ink structures of the upper sub-metasurfaces of the two unit cells are different (the upper layer of unit cell 1 has no resistive ink, while the upper layer of unit cell 2 has a square structure). The dielectric material is flexible sponge (with a relative permittivity $\varepsilon_{r}$ ≈ 1.1 in the microwave frequency band) to ensure the flexibility of the metasurface; the bottom layer of both unit cells is provided with a perfect electric conductor (PEC) to form a reflective structure.

The thickness of the resistive ink is negligible, and it is printed on a flexible cloth with a thickness of 0.13 mm (relative permittivity $\varepsilon_{r}$ ≈ 3) in the microwave frequency band. Therefore, in the CST simulation, the cloth thickness is set to 0.13 mm and the relative permittivity to 3. The unit size parameters and sheet resistance values are optimized using CST (CST—Computer Simulation Technology AG, Darmstadt, Germany) software. When $R_{SD1}$ = $R_{SD2}$ = 35 Ω/sq, $R_{SU1}$ = 350 Ω/sq, h = 6 mm, p = 5 mm, $a_{1}$ = 4.7 mm, $a_{2}$ = 3.9 mm, and b = 4.7 mm, unit cell 1 and unit cell 2 can have different reflectivity amplitudes and a large reflectivity phase difference. The simulation results are shown in Figure 2c,d.

Both unit cell 1 and unit cell 2 have resonant frequencies in the 2–18 GHz band: unit cell 1 has a resonant point at 8 GHz, with a small reflectivity amplitude and a wide absorption bandwidth around the resonance; unit cell 2 has one resonant point each at 4 GHz and 16 GHz. The resonance at 4 GHz is sharp with a small reflectivity amplitude but a narrow local bandwidth, while the resonance at 16 GHz is gentle with a large reflectivity amplitude. The superposition of these two resonances enables unit cell 2 to achieve a reflectivity of <−10 dB over a broadband range. To further reduce the energy of incident waves from SAR for imaging cloaking, it is necessary to reduce the reflectivity of the metasurface through specific array arrangement. As shown in Figure 2d, the phase difference between the two unit cells is over 120° in the 4.4–16 GHz band, and especially close to 180° in the 8–12 GHz band. Combined with the difference in their resonant frequencies and the large phase difference over a wide frequency band, according to the far-field radiation theory, after specific array arrangement, the reflectivity of the metasurface can be further reduced by 5–10 dB on the basis of the original unit cells (for example, the original reflectivity of unit cell 2 is approximately −10 dB, which can reach −15 to −20 dB after array arrangement), thereby improving the broadband absorption performance as a whole.

**Figure 3:**
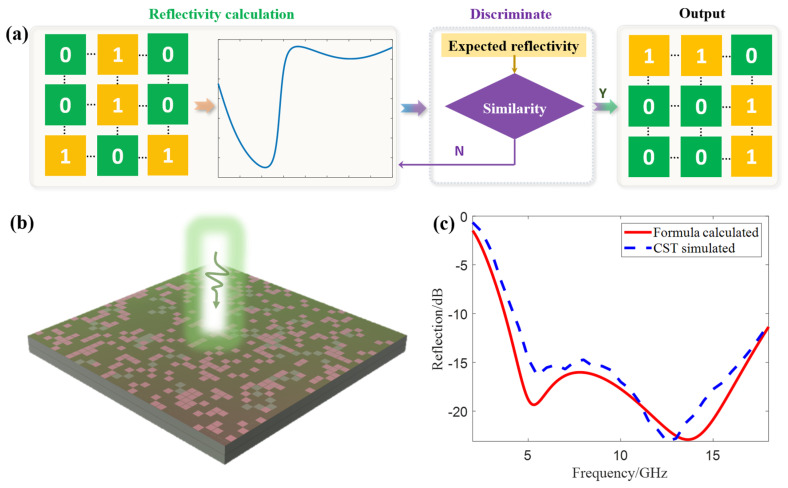
Inverse solution process of the metasurface array. (**a**) Flowchart for designing the array metasurface. (**b**) The designed metasurface. (**c**) Comparison between theoretical calculation and simulation results.

Figure 3a shows the design flowchart of the array metasurface: first, the expected wave absorption performance is specified (such as the reflectivity threshold at specific frequency points/bands); a random permutation matrix containing only 0 s and 1s is generated via MATLAB (It is developed by MathWorks, Inc., located in Natick, MA, USA, and has been purchased by Zhejiang University); and the reflectivity of the array metasurface corresponding to the permutation matrix is calculated using the far-field formula, and the result is compared with the expected reflectivity. If the expected performance is not met, the permutation matrix is discarded and a new one is regenerated until a permutation matrix that meets the expected performance is obtained.

Based on the above process, combined with the subsequent X-band SAR test requirements, the performance indicators of the array metasurface are set as follows: the reflectivity in the 8–12 GHz band is <−15 dB (since the reflectivity of unit cell 2 in this band is <−10 dB, and the reflectivity of unit cell 1 is lower, which can be further reduced after array arrangement); at the same time, it is necessary to ensure high absorption rate in a wide band. Considering that the reflectivity of unit cell 1 in low and high frequencies is >−10 dB, it is required that the reflectivity at 4 GHz and 18 GHz is <−10 dB.

According to the performance indicators, MATLAB is used to write codes for generating array matrices and calculating far-field reflectivity. The size of the array is determined by the size of the experimental sample (to save costs, the sample size is 180 mm × 180 mm, and the array size is 36 × 36). Since the randomly generated 36 × 36 matrix usually has no symmetry and has difficultly coping with full-polarization SAR, the matrix is modified to 18 × 18, and symmetric processing about the x-axis, y-axis and origin is performed in the plane respectively. Then, it is spliced with the original matrix to obtain a 36 × 36 symmetric array matrix (satisfying symmetry about the x-axis, y-axis and rotational symmetry).

The reflectivity of several array matrices is calculated and compared with the expected indicators, and finally, an array matrix that meets the requirements is obtained. The corresponding metasurface is shown in Figure 3b, and the comparison between theoretical calculation and simulation results is shown in Figure 3c. The results show that the array metasurface exhibits two resonance points (5.3 GHz and 13.65 GHz) in the 2–18 GHz band, with reflectivity of −19.3 dB and −22.9 dB respectively. The resonance points are gentle with a large local absorption bandwidth, and the reflectivity is low in a wide frequency band; the reflectivity in the 8–12 GHz band is <−15 dB, and the reflectivity at 4 GHz and 18 GHz are −11.9 dB and −11.4 dB respectively, all meeting the expected indicators. It should be noted that the near-field electromagnetic coupling between adjacent unit cells modifies the local field distribution, leading to a shift in the resonance frequencies. Specifically, the aperiodic arrangement of the two unit cells introduces new electromagnetic interaction modes, causing the 3 GHz and 8 GHz resonances of the isolated unit cells to evolve into the 5 GHz and 13 GHz reflection dips of the array under collective action. This phenomenon conforms to the general rule of “array coupling modulating resonance frequencies” in metasurface design.

The resistive ink needs to be printed on a flexible fabric with a thickness of 0.13 mm, which has a relative permittivity of approximately 3 and a loss tangent of 0.0002 in the microwave frequency range. The resistive ink is a paste made by mixing conductive materials such as metal particles or carbon-based materials with organic resins and solvents in a certain proportion. It is printed on flexible fabric via inkjet printing. For the entire metasurface, the resistance value of the top resistive ink layer is 350 Ω/sq and that of the second layer is 35 Ω/sq. To ensure the flexibility of the metasurface, a flexible sponge is used to isolate the two layers of flexible fabric. The prepared flexible metasurface samples are tested in a microwave anechoic chamber at room temperature (around 25 °C), using a 1–40 GHz vector network analyzer as the source signal and a broadband horn antenna for transmission and reception.

**Figure 4:**
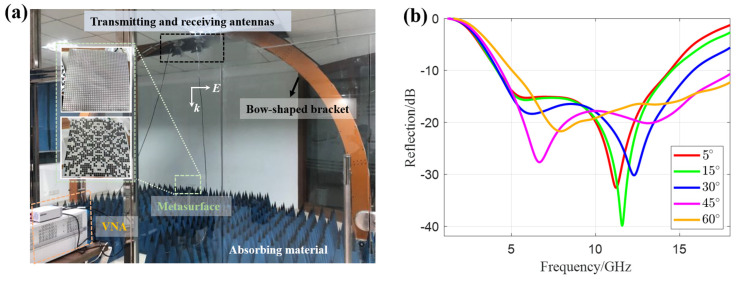
Indoor experimental results. (**a**) Experimental setup. (**b**) Reflectivity curves under different incident angles.

In a microwave anechoic chamber, a bow-frame structure was used to conduct bistatic far-field reflectivity tests on the sample (the experimental setup is shown in Figure 4a, with the inset being the flexible metasurface sample. The experimental equipment and devices shown in Figure 4a were purchased from Qingdao 41st Institute, and the samples shown were processed by Fujian Haichuan Plastic & Rubber Co., Ltd. All of them are currently stored in Hangzhou, Zhejiang Province). The transmitting and receiving antennas were controlled by a slide rail, so that the incident angle was equal to the receiving angle. The reflectivity at incident angles of 0–60° was tested (Figure 4b). At normal incidence, the metasurface had two resonance points (5.6 GHz and 11.2 GHz) in the 2–18 GHz band. As the incident angle increased, the resonance points shifted to higher frequencies. At an incident angle of 45°, the bandwidth with reflectivity <−10 dB was the largest (4.37–18 GHz). At an incident angle of 60°, the bandwidth was reduced to 5–18 GHz. At normal incidence, the bandwidth was 4.16–14.4 GHz. The results show that the flexible metasurface has the optimal wave absorption performance at a 45° oblique incidence. Since the oblique incidence angles of EM waves from detection systems such as radars are usually 45–60°, this metasurface is suitable for SAR imaging cloaking tests.

**Figure 5:**
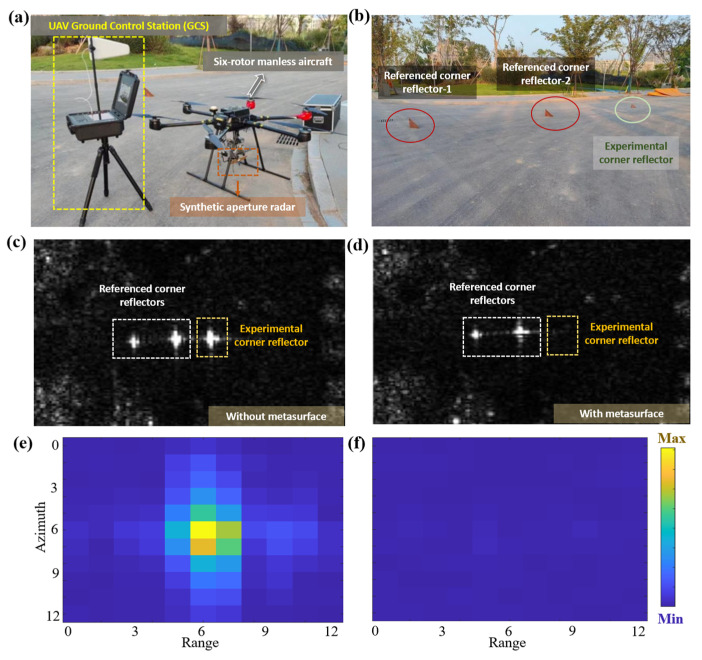
UAV-borne SAR experimental results. (**a**) UAV-borne SAR equipment. (**b**) Three corner reflectors used in the experiment. (**c**) SAR imaging result without the metasurface loaded. (**d**) SAR imaging result with the metasurface loaded. (**e**) Magnified view of the imaging area of the corner reflector without the metasurface loaded. (**f**) Magnified view of the imaging area of the corner reflector with the metasurface loaded.

To verify the cloaking performance of the flexible metasurface in SAR imaging, an outdoor test was carried out using a UAV platform (The experimental equipment and devices shown in Figure 5a were purchased from “Xi’an Sine Wave Measurement and Control Technology Co., Ltd” and are currently located in Hangzhou, Zhejiang Province, China). Figure 5a shows the outdoor test environment and system equipment (including UAV-borne SAR equipment, ground base station, and remote-control terminal). The UAV-borne SAR system used in the outdoor experiments of this study operates in the X-band, with a center frequency of 9.6 GHz, a bandwidth of 1800 MHz, and a resolution better than 0.3 m. The UAV ground base station is responsible for controlling the take-off, landing, and route setting of the UAV. The X-band radar is suspended under the UAV, and a laptop is used for remote control of radar scanning. The test scenario is an open asphalt road, on which three corner reflectors are placed (Figure 5b). The one marked with a red circle is the calibration corner reflector, and the one marked with a green circle is the corner reflector to be cloaked. Considering that the resolution of the X-band radar is 0.167 m, to ensure clear imaging without mutual interference, the distance between the corner reflectors is greater than 1 m. The asphalt road has a diffuse reflection characteristic for EM waves, showing a dark area in SAR imaging; the corner reflector can return most of the EM wave energy to the radar in the form of echo, thus appearing as a bright spot in SAR imaging, which is often used for positioning and calibration in SAR imaging.

The cloaking performance of the metasurface was quantified through a comparative experiment: first, none of the three corner reflectors was loaded with the metasurface, and the flight mission was carried out according to the set route for SAR scanning; then, only the corner reflector marked with the green circle was loaded with the metasurface, and the flight and scanning tasks were repeated. The cloaking effect was evaluated by comparing the brightness of the corner reflectors in the two images. Figure 5c,d show the SAR imaging results of the two experiments, and Figure 5e,f show the magnified views of the corresponding imaging areas of the corner reflectors. The results show that the imaging brightness of the corner reflector is significantly reduced after loading the metasurface, indicating that the metasurface can effectively reduce the echo energy of the object and has SAR imaging cloaking performance.

To quantitatively describe the cloaking effect, the structural similarity (SSIM) of the imaging areas in Figure 5e,f was calculated. The results show that the image similarity before and after cloaking is only 0.9%, confirming that the designed flexible metasurface has excellent SAR imaging cloaking effect.

## 3. Conclusions

We designed and verified a broadband SAR imaging flexible invisibility metasurface via multi-domain joint regulation, addressing the application limitations of traditional rigid invisibility devices in complex scenarios. The two proposed unit cells, with differentiated amplitude/phase characteristics (≈180° phase difference), achieve > 10 dB RCS reduction in 2–18 GHz after array arrangement; optimized via inverse design, it exhibits <−15 dB reflectivity in 8–12 GHz (X-band) and <−10 dB at 4/18 GHz, meeting broadband indicators. Indoor experiments confirm stable absorption under oblique incidence (−10 dB bandwidth: 4–18 GHz), and outdoor SAR imaging shows significantly reduced target brightness (SSIM: 0.9%), verifying its excellent invisibility. Practically, it provides a feasible solution for dynamic EM protection of ground temporary facilities and SAR anti-detection. It should be noted that for potential applications in scenarios such as aircraft, marine structures, and military equipment, subsequent targeted environmental adaptability verification will be required in combination with specific usage environments, including performance evaluation under conditions such as high-low temperature humidity, ultraviolet radiation, and salt spray to ensure its stability in complex environments. Future work may focus on optimizing flexible substrate stability and expanding multi-scenario adaptability.

## Data Availability

The original contributions presented in this study are included in the article. Further inquiries can be directed to the corresponding authors.
